# Very-low and low-density lipoproteins induce neutral lipid accumulation and impair migration in monocyte subsets

**DOI:** 10.1038/srep20038

**Published:** 2016-01-29

**Authors:** William D. Jackson, Tobias W. Weinrich, Kevin J. Woollard

**Affiliations:** 1 Division of Immunology and Inflammation, Department of Medicine, Imperial College London, UK; 2 Department of Visual Neuroscience, Institute of Ophthalmology, University College London, UK.

## Abstract

Blood monocytes are heterogeneous effector cells of the innate immune system. In circulation these cells are constantly in contact with lipid-rich lipoproteins, yet this interaction is poorly characterised. Our aim was to examine the functional effect of hyperlipidaemia on blood monocytes. In the *Ldlr*^*−/−*^ mouse monocytes rapidly accumulate cytoplasmic neutral lipid vesicles during hyperlipidaemia. Functional analysis *in vivo* revealed impaired monocyte chemotaxis towards peritonitis following high fat diet due to retention of monocytes in the greater omentum. *In vitro* assays using human monocytes confirmed neutral lipid vesicle accumulation after exposure to LDL or VLDL. Neutral lipid accumulation did not inhibit phagocytosis, endothelial adhesion, intravascular crawling and transmigration. However, lipid loading led to a migratory defect towards C5a and disruption of cytoskeletal rearrangement, including an inhibition of RHOA signaling. These data demonstrate distinct effects of hyperlipidaemia on the chemotaxis and cytoskeletal regulation of monocyte subpopulations. These data emphasise the functional consequences of blood monocyte lipid accumulation and reveal important implications for treating inflammation, infection and atherosclerosis in the context of dyslipidaemia.

Monocytes are a heterogeneous, key population of the mononuclear phagocyte system that fulfil a variety of innate immune functions and have independent phenotypes from their polarised macrophage descendants^1^. At least two functionally distinct monocyte populations exist in mammals including humans, mice, rats, pigs and cows^2^. Monocyte populations can be defined based on their expression of Ly6C/GR1 in mice and CD14 or CD16 expression in humans. The Ly6C/GR1-high (GR1^hi^) and homologous CD16-negative CD14-high (CD16^neg^) population are ‘classical’ monocytes, which can be recruited to inflamed tissue and respond strongly to bacteria stimuli. Whereas the Ly6C/GR1-low (GR1^low^) and homologous CD16-positive CD14-low (CD16^pos^) monocytes are ‘non- classical’, and respond to viral and TLR7/8 cues, and have been shown to patrol the endothelium^3^. A third, ‘intermediate’ population is reported in humans^3^,^4^,^5^ which express high levels of both CD14 and CD16. This population can be expanded in inflammatory disease^6^, but typically represent a minor population of total monocytes and clusters transcriptionally with the ‘non-classical’ group^4^. Therefore to align broadly with the two ‘classical’ and ‘non-classical’ populations in mice, human monocytes can be defined as 2 subsets based on CD16 expression, as previously described^7^,^8^.

The synthesis, processing, transport and catabolism of circulating lipid species is complex and involves many different cell types and metabolic processes^9^. Hypercholesterolemia and dyslipidaemia with either elevated LDL or VLDL has a strong association with cardiovascular disease progression^10^,^11^ and therefore atherosclerosis is the predominant focus in dyslipidaemia research. However the direct role of dyslipidaemia in immunity remains enigmatic. Human epidemiological data demonstrates that general surgery patients in the 5th or 95th percentile of total blood cholesterol levels have an approximate 4.3-fold increased risk of hospital-acquired infection^12^. Moreover, experimental models of dyslipidemia with elevated levels of VLDL and LDL lead to immune suppression during infection with Staphylococcus aureus, Mycobacterium tuberculosis, Candida albicans and Listeria monocytogenes, resulting in increased pathogen load and defective phagocytosis^13^,^14^,^15^,^16^. Together these findings strongly suggest a possible inhibitory effect of dyslipidemia on monocytes and macrophages in infection and pro-atherosclerotic effects in cardiovascular disease.

Previous work on lipoprotein biology and the mononuclear phagocyte system has mainly focused the role of LDL on macrophages and dendritic cells (DC) in atherosclerosis progression^17^. However, circulating lipophages have been described *in-vivo* since the 1960s, which are most likely neutral lipid positive blood monocytes after high fat feeding^18^,^19^,^20^,^21^,^22^,^23^,^24^. More recently, it has been shown that these ‘foamy’ monocytes can enter early atherosclerotic plaques^25^ and hypertriglyceridemia can mediate non-classical margination and macrophage tissue accumulation^26^. Together these data raise the possibility that dyslipidaemia can affect monocyte phenotype and possibly functionality. Therefore given the increasing disparity between monocytes and tissue macrophages^1^ and the distinct kinematic phenotypes of monocyte subpopulations in homeostatic and inflammatory conditions during atherosclerosis^25^, further investigation was warranted into the effects of dyslipidaemia on monocyte migration during inflammation independent of cardiovascular disease.

We demonstrate here that monocytes accumulate cytoplasmic neutral lipid droplets in response to LDL and VLDL, which subsequently alters their cytoskeletal dynamics both *in vitro* and in the hypercholesterolemic *Ldlr*^*−/−*^ mouse. Strikingly, the extravascular chemotaxis of monocytes is impaired by lipid accumulation, in part mediated by RHOA inactivation. These findings underscore the functional role of blood monocytes and suggest that dyslipidaemia associated with monocyte neutral lipid accumulation, may result in monocyte immunosuppression.

## Materials and Methods

### See extended methods in supplemental information

All animal procedures were carried out according to the Institutional guidelines for the care and use of experimental animals and the ARRIVE guidelines. Animal studies were approved by the UK Home Office. Blood from healthy human donors was collected under institutional guidelines with informed consent approved by NRES Committee London.

### Peritonitis model

*Ldlr*^*−/−*^ mice were maintained on HFD or chow for 16 weeks. To induce peritonitis, mice were injected intraperitoneally (IP) with 1ml sterile 4% thioglycollate medium. After 72 hours, mice were culled and the peritoneal cavity was lavaged with 10 ml ice-cold PBS. Approximately 3 × 10^5^ cells were stored in Tri-Reagent (Sigma Aldrich) for RNA extraction, the remainder were stained in PBS-0.5% BSA for flow cytometric analysis in a saturating concentration of anti-CD16/32 (2.4G2) using combinations of the following antibodies: anti-CD115 (AF598), anti-CD11c (N418), anti-CD45 (30-F11) (eBioscience), anti-CD11b (M1/70), anti-GR1 (RB6-8C5) anti-F4/80 (BM8) (BD Biosciences). Monocytes were defined as CD115^pos^ CD11b^pos^ SSC^int^ (see supplemental Figure 2b). In some experiments beads were used to track extravasation and omentum was collected to analyse monocyte infiltration.

### Monocyte purification

Human monocyte subsets were purified from healthy donors using Monocyte Isolation Kit II and anti-CD16 microbeads (Miltenyi). Mouse monocytes were purified using FACS. Surface protein expression was assessed using mouse antibodies (as above) and human HLA-DR (TU36), CD16 (3G8), CD11c (Bu15) and CD14 (M5E2) (BD Biosciences).

### Fluorescence microscopy

Cells were stained for neutral lipid using LipidTox-Green (Life Technologies). To image the actin cytoskeleton cells were stained with phalloidin- AlexaFluor 488 (Life Technologies). Mounted cells were imaged using either a Zeiss AxioObserver widefield or a Leica SP5 confocal microscope with a 63×/1.4 objective. Images were analysed using Imaris software (Bitplane) or ImageJ, as indicated.

### Monocyte adhesion and migration

Purified monocyte subsets were analysed for migration using transwells (Corning) and 2D-chemotaxis chambers (IBIDI). Adhesion and transendothelial migration (TEM) was assessed in static co-cultures using transwells and chamber slides (IBIDI). Intravital imaging of monocyte intravascular migration was assessed in high fat fed *Cx3cr1*^gfp/+^ mice as previously described^27^.

### Monocyte phagocytosis and cell death

Phagocytosis was examined using fluorescence latex bead (Life Technologies) and cell death examined using Annexin V (eBioscience).

### Cytoskeletal signaling

Purified monocytes were treated with LDL and VLDL (100 μg/ml) for 2 hours and lysates collected for RHOA activation using luminescence-based G-LISA RhoA activation kit according to manufacturer instructions (Cytoskeleton, Inc.). CDC42 activity was assessed using colorimetric-based G-LISA kit according to manufacturer instructions (Cytoskeleton, Inc.) and PAK activity was assessed using anti-phospho-PAK1/2/3 (Novus Biologicals) via Western blot.

### Statistics

Experimental data is presented as mean +/− standard error of the mean (SEM). Populations were compared using a two-tailed Mann–Whitney U test to avoid assumptions of parametric distribution. P < 0.05 was considered significantly different.

## Results

### *In vivo* phenotyping of monocyte subsets in response to hyperlipidaemia

**Figure 1:**
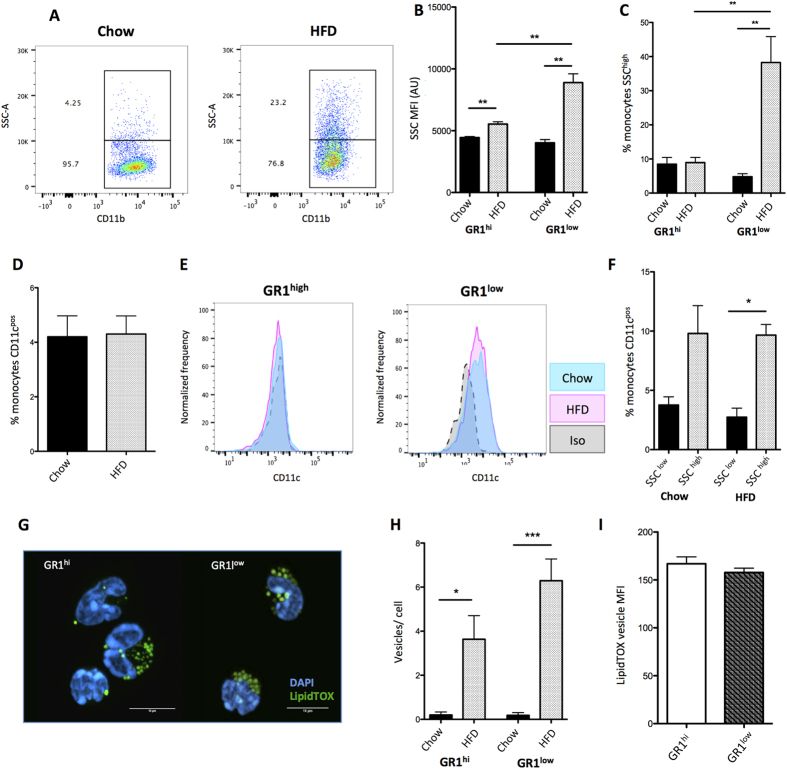
Blood monocytes from *Ldlr*^*−/−*^ mice are lipid loaded. Blood monocytes from *Ldlr*^*−/−*^ mice fed chow or high fat diet (HFD) for 8 weeks were analysed by flow cytometry. (**A**) A representative FACS dot plot example of SSC^high^/SSC^low^ monocytes on chow and after HFD, gated from CD115^pos^ cells. (**B**) Quantification of GR1^high^ and GR1^low^ monocyte SSC with (grey bars) or without HFD (black bars). (**C**) Percentage of GR1^high^/GR1^low^ monocytes that are SSC^high^ on chow/HFD. (**D**) Percentage of monocytes that are CD11c^pos^ on chow or HFD, gated from CD115^pos^ CD11b^pos^ cells. (**E**) Representative histogram of GR1^high^ or GR1^low^ monocyte CD11c expression, gated from CD115^pos^ CD11b^pos^ cells. (**F**) Percentage of SSC^high^/SSC^low^ monocytes that are CD11c^pos^ on chow or HFD. (**G**) Neutral lipid staining of Ly6c^hi^ and Ly6c^low^ blood monocytes after 16 weeks HFD. Scale bar represents 10 μm. Staining is quantified as (**H**) vesicles per cell (20–25 cells per condition) and (**I**) LipidTOX vesicle median fluorescence intensity (MFI) (20–25 cells per subset) in HFD only. Error bars show the mean±SEM. *, ** and *** represents P < 0.05, P < 0.01 and P < 0.001 respectively analysed by Mann–Whitney U test. n = 3–4 mice per group. See also Sup. Fig. 1.

We aimed to characterise the interactions of monocyte subsets (GR1^hi^ and GR1^low^) with blood lipoproteins in the hyperlipidaemic *Ldlr*^*−/−*^ mouse with raised plasma VLDL and LDL^28^. When analysed by flow cytometry, monocyte side-scatter (SSC) was significantly elevated in both subsets after 8 weeks HFD when compared to chow, indicating increased cell granularity (Fig. 1a,b), predominantly on GR1^low^ monocytes (Fig. 1c). Monocyte CD11b levels decreased on both subpopulations on HFD (Supplemental Fig. 1a), while MHC-II (I-A) and GR1 expression did not change (Supplemental Fig. 1b,c) indicating no monocyte activation as measured by these surface protein markers. However, previous work has shown an increase in CD11c during hyperlipidaemia^22^,^25^. In our hands, only 4% of monocytes expressed CD11c, mainly on GR1^low^, which was not elevated after high fat diet as percentage of cells or MFI (Fig. 1d,e). However, there was a modest but significant increase in percentage of monocytes expressing CD11c in SSC^hi^ monocytes from both chow and HFD *Ldlr*^*−/−*^ mice (Fig. 1f). More recently others have reported CD36 expression to indicate CD11c+ ‘foamy monocyte’ during HFD in *ApoE*^*−/−*^ mice^25^, therefore we also examined CD36 expression. While 80–85% of all monocytes expressed CD36, there was no change in expression after HFD or between subsets in the *Ldlr*^*−/−*^ mouse (Supplemental Fig. 1d). Finally, GR1^hi^ neutrophils showed no change in granularity or CD11b expression (Supplemental Fig. 1e,f).

To investigate the composition of this increased monocyte granularity, we sorted (gating strategy Supplemental Fig. 2a) blood monocytes from the *Ldlr*^*−/−*^ mouse, stained for neutral lipid using the lipophilic fluorescent dye LipidTOX and visualized using confocal microscopy. HFD causes a marked accumulation of cytoplasmic neutral lipid vesicles in both monocyte subsets (Fig. 1g). This was true for approximately 70% of monocytes from animals on HFD and no such vesicles were observed on a chow diet (data not shown). Further analysis demonstrated that HFD causes a significant increase in the number of neutral lipid vesicles per cell in both monocyte subsets (Fig. 1h). There were no significant differences in lipid accumulation between monocyte subsets when analysed for SSC or mean LipidTOX vesicle intensity (Fig. 1b,i).

### HFD impairs monocyte accumulation in the inflamed peritoneum due to retention in the omentum

**Figure 2:**
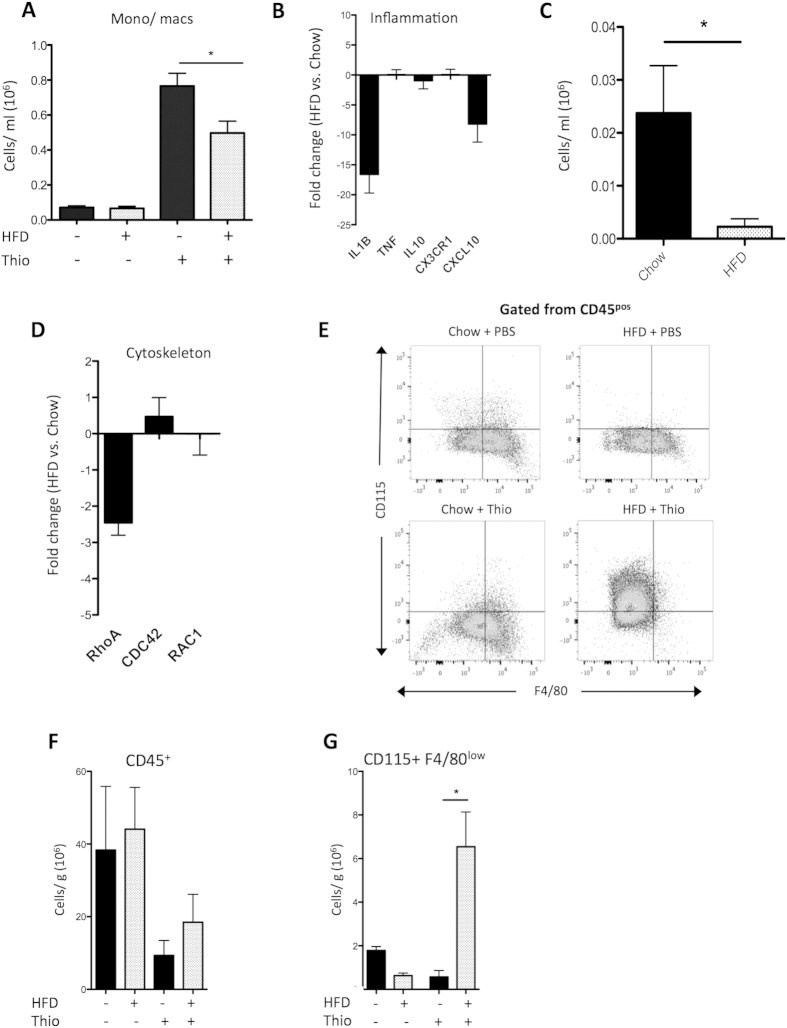
Sterile peritonitis model in dyslipidemic *Ldlr*^*−/−*^ mice. Thioglycollate peritonitis (72 hours post-thioglycollate injection; Thio) or controls was induced in *Ldlr*^*−/−*^ mice (n = 4 per group) with or without 16 week high fat diet (HFD) and peritoneal lavage collected. (**A**) Monocytes and macrophages/ml gated as CD115+ CD11b+ (**B**) Inflammatory gene expression in peritoneal cells from Thio peritonitis (fold change in HFD versus chow). (**C**) Intravenous injection of 1 μm latex beads was used to track monocyte migration out of the blood during peritonitis. CD115^+^CD11b^+^Bead^+^ cells/ml in peritoneal lavage from *Ldlr*^*−/−*^ mice with or without HFD (n = 4 per group). (**D**) Cytoskeletal gene expression in peritoneal cells from Thio peritonitis (fold change in HFD versus chow). (**E**) Representative flow cytometry plots of CD115 and F4/80 staining in the omentum of *Ldlr*^*−/−*^ 72 hours post Thio, gated from all CD45+ cells. (n = 4–5 mice per group). (**F-G**) Leukocyte populations/gram of tissue from (**E**): (**F**) CD45^+^ leukocytes, (**G**) CD115+ F4/80^low^ monocytes. * and ** represents P < 0.05 and P < 0.01 respectively analysed by Mann–Whitney U test. See also Sup. Fig. 2.

To investigate the functional impact of blood monocyte lipid loading on their response to inflammation, we performed thioglycollate peritonitis in the *Ldlr*^*−/−*^ mouse and FACS analysed cells from the peritoneal lavage at 72 hours post-injection; a time point well characterized to represent pronounced monocyte/macrophage infiltration^29^. (FACS gating strategy for peritoneal monocyte/macrophages is shown in Supplemental Fig. 2b). Thioglycollate (Thio) increased total CD11b+CD115+ monocyte/macrophages in the peritoneum and this was significantly decreased by HFD (Fig. 2a). As expected^29^, very few (20-fold less than monocytes) CD11b+CD115^neg^GR1^high^ granulocytes were found in the peritoneum at 72 hours (Supplemental Fig. IIc). To further characterize this phenotype, we assessed gene expression by qPCR of cells from the peritoneum following thioglycollate peritonitis. As previously observed by others^30^, these cells exhibited a ‘de-activated’ phenotype characterized by decreased expression of inflammatory genes *Il1β* and *Cxcl10* after HFD (Fig. 2b).

We hypothesized that neutral lipid loading may be causing a chemotactic defect in blood monocytes, based on previous work in macrophage foam-cells^31^,^32^. To determine any blood monocyte migratory defect, we labelled blood phagocytes using intravenous (IV) injection of fluorescent latex beads, as previously reported^19^. As expected, bead injection labelled both subsets, with some preference for GR1^low^ monocytes (Supplemental. Fig. 2d). Thioglycollate peritonitis was induced 4 hours after bead injection, and peritoneal lavage analysed at 72 hours. While bead fluorescence intensity was equal in chow and HFD mice (Supplemental Fig. 2e), indicating that phagocytosis was not impaired, there was a significant decrease in the number of bead-positive CD115+ monocytes in the peritoneum (Supplemental Fig. 2f,g). We therefore concluded that blood monocytes exposed to a HFD have an intrinsic migratory defect. In keeping with this hypothesis, peritoneal cells from HFD mice had decreased expression of the cytoskeletal small GTPase *RhoA*, which is required for efficient leukocyte chemotaxis (Fig. 2d)^32^,^33^. Interestingly, we also noted decreased expression of *RhoA* in published microarray data from monocytes of patients with familial hypercholesterolemia, supporting an effect of hypercholesterolemia on cytoskeletal regulation^34^ (NCBI GEO GSE6054, data not shown).

At this stage, it was unclear whether HFD monocytes were failing to extravasate into the sub-endothelial space, or whether they were being retained in tissue before reaching the peritoneal cavity. Previous work has demonstrated that the greater omentum is the major site of leukocyte extravasation during rodent peritonitis^35^, therefore we harvested and analysed the greater omentum by FACS at 72 hours post- thioglycollate peritonitis to assess the number of leukocytes (Fig. 2e). On both chow and HFD, peritonitis decreased the number of CD45+ leukocytes in the omentum (Fig. 2f), as macrophages migrate from reserves in the omental ‘milky spots’ into the peritoneal cavity. However, HFD causes an approximate 10-fold increase in the number of CD115+ F4/80^low^ monocytes in the omentum during peritonitis (Fig. 2g). These data provide strong evidence that the decreased accumulation of monocytes/macrophages in the peritoneum on high fat diet is due to blood monocyte retention in the omentum following extravasation.

### Human monocytes accumulate cytoplasmic neutral lipid in response to LDL or VLDL

**Figure 3:**
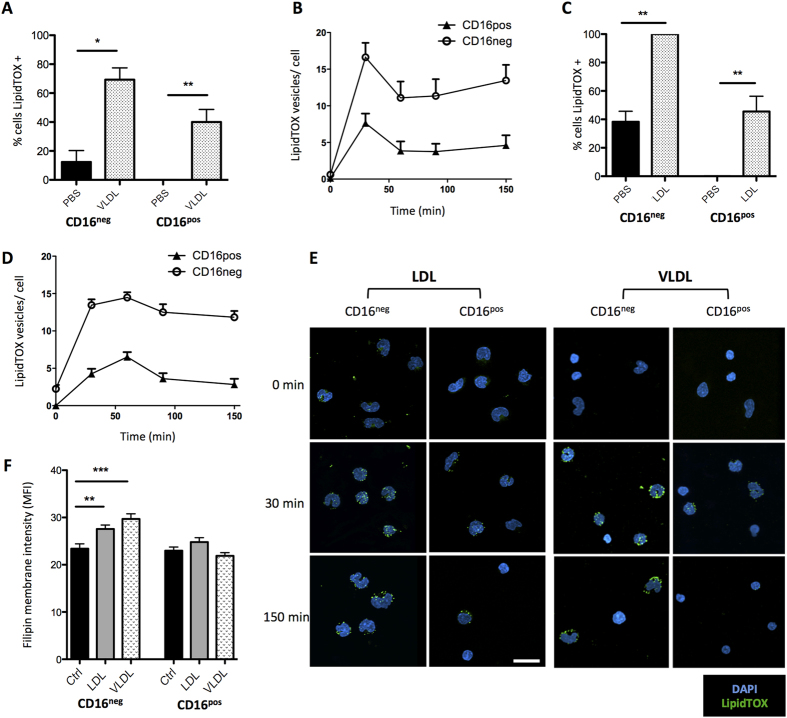
Neutral lipid accumulation in LDL and VLDL treated monocytes. Human CD16^pos^ and CD16^neg^ monocytes were purified and treated with LDL or VLDL (100 μg/ml) and intracellular neutral lipid was quantified using LipidTOX by confocal microscopy. (**A**) Percentage cells neutral lipid positive after 150 mins VLDL treatment (n = 10 fields). (**B**) Number of neutral lipid vesicles per cell over time after VDL treatment (n = 25–50 cells). (**C,D**) Same as (**A**,**B**) respectively with LDL treatment. (**E**) Representative examples of neutral lipid positive monocytes from LDL and VLDL treatments. Scale bar represents 20 μm. (**F**) Membrane cholesterol content in monocytes after 120 mins LDL or VLDL treatment, quantified by filipin staining intensity. Error bars show the mean ± SEM. *, ** and *** represents P < 0.05, P < 0.01 and P < 0.001 respectively analysed by Mann–Whitney U test. See also Sup. Fig. 3.

Following observations in the *Ldlr*^*−/−*^ mouse, we attempted to recapitulate the effects of hypercholesterolemia on human monocytes *in vitro*. It is well established that VLDL is the blood lipoprotein fraction most elevated by HFD and most abundant in the *Ldlr*^*−/−*^ mouse^28^,^36^. As such we specifically investigated the effects of VLDL, which is often overlooked in *in vitro* studies, as well as further characterizing the effects of LDL. When exposed to 100 μg/mL VLDL for 150 minutes, both monocyte subsets accumulated neutral lipid vesicles, although vesicles were present in approximately 70% of CD16^neg^ cells and 45% of CD16^pos^ (Fig. 3a). When quantified as number of lipid vesicles per cell over time, peak accumulation was at 30 minutes, with CD16^neg^ monocytes containing approximately 15 lipid vesicles per cell and CD16^pos^ approximately half this number (Fig. 3b). A similar phenotype was seen with LDL treatment, although peak lipid accumulation was delayed to 60 minutes (Fig. 3c,d) and showed a modest increased number of cells contained neutral lipid vesicles overall (compared to VLDL). Representative images are shown in Fig. 3e, and are visually similar to blood monocytes from the *Ldlr*^*−/−*^ mouse on HFD (Fig. 1g).

Previous work examining macrophages from ABCA/G1 knockout mice, but not acLDL treated cells, demonstrated an increased membrane cholesterol content that was suggested to be responsible for a migratory defect^31^. Therefore we analysed at membrane cholesterol content using filipin. CD16^neg^ monocytes showed a modest but significant 10–20% increase in filipin staining after LDL or VLDL treatment, which was not seen in CD16^pos^ cells (Fig. 3f). However, given that neutral lipid accumulation increased 60–70% in both subsets after lipid treatments, we concluded that VLDL and LDL treatment predominantly increases neutral lipid content in all monocytes. Despite this striking neutral lipid accumulation, we observed no effect of VLDL or LDL on CD11c, HLA-DR, CD14 or CD16 surface expression, potential markers of monocyte activation (Supplemental Fig. 3a–h). Moreover, there was no change in cell death or phagocytosis (Supplemental Fig. 3i–l).

### Lipid accumulation impairs monocyte extravascular migration

**Figure 4:**
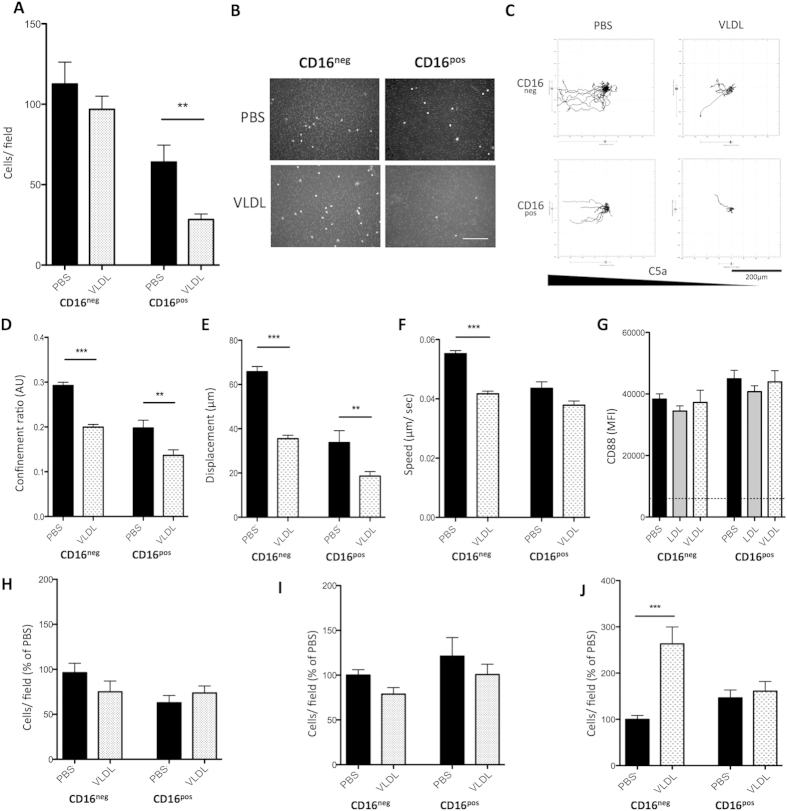
Effects of VLDL treatment on monocyte migration. (**A**) Transwell migration of CD16^pos^ and CD16^neg^ monocytes to C5a (250 ng/ml) with or without VLDL pre-treatment (2 hrs; 100 μg/ml). Data represents 12 fields of view from 3 independent experiments. (**B**) Representative cells/field migrated shown in (**A**) (scale bar = 100 μm). (**C**) Representative track projections from CD16^pos^ and CD16^neg^ monocytes with or without VLDL pre-treatment (2 hrs; 100 ug/ml) in a 2D real-time chemotaxis assay towards a C5a gradient (n = 3 independent experiments). Tracks were analysed for (**D**) confinement ratio (**E**) displacement (μm) and (**F**) speed (μm/sec) (n = 120–800 cells) (**G**) CD16^pos^ and CD16^neg^ cell surface expression of CD88 after 2 hours LDL or VLDL treatment, assessed by flow cytometry (n = 2 donors in duplicate). (**H**) Adhesion of CD16^pos^ and CD16^neg^ monocytes to HUVECS from only monocytes pre-treated with VLDL (2 hrs; 100 μg/ml), normalised to CD16^neg^ PBS treated (n = 4 donors), (**I**) Same as (**H**) with only HUVECS treated with VLDL. (**J**) Transmigration of CD16^pos^ and CD16^neg^ monocytes pre-treated with (2hrs; 100 μg/ml) or without VLDL through TNF activated HUVEC: cells per field, normalised to CD16^neg^ PBS treated. (n = 4 donors) Error bars show the mean ± SEM. *** represents P < 0.001 analysed by Mann–Whitney U test. See also Sup. Fig. 4.

Given the reduced accumulation of mouse monocytes following peritonitis on HFD, we next investigated whether neutral lipid loading would impair monocyte chemotaxis *in vitro*. Using transwell chambers, we found that pre-treatment with 100 μg/mL VLDL inhibited monocyte chemotaxis towards C5a, mainly in CD16^pos^ monocytes (Fig. 4a,b). A similar effect was seen with LDL treatment (Supplemental Fig. 4a). To examine any defect in migration in greater detail, we utilized a more realistic C5a chemotactic model using real-time microscopy with 2D migration chambers. VLDL treated CD16^neg^ and CD16^pos^ monocytes showed decreased track displacement and confinement ratio, accompanied by an almost complete failure to polarize towards the chemoattractant (Fig. 4c–e). Moreover, VLDL treatment decreased migratory speed towards C5a (Fig. 4f), all indicative of chemotactic inhibition after VLDL treatment, which did not appear to be subset specific. Interestingly, this migratory inhibition also existed during monocyte chemokinesis, when the chemoattractant was uniformly distributed and the cells were not required to polarize (Supplemental Fig. 4b–e). Importantly, to examine if the defect in C5a induced migration was simply an effect of VLDL on the C5a receptor (CD88), we examined CD88 expression levels before and after lipid treatments. CD88 expression was not significantly different between monocyte subsets or after VLDL treatment (Fig. 4g), indicating that the defect in migration was not a reduction in C5a receptor signaling.

We next examined monocyte-endothelial interactions after lipid treatments. Monocyte adhesion to a HUVEC endothelial layer was unchanged by VLDL treatment of the monocytes (Fig. 4h) or the endothelium (Fig. 4i). However, LDL treatment did increase CD16^neg^ endothelial adhesion, as reported by others^37^ (Supplemental Fig. 4f,g). There was no effect of VLDL on CD16^pos^ migration across a HUVEC monolayer, although VLDL did increase CD16^neg^ monocyte trans-endothelial migration (TEM) (Fig. 4j). A similar effect was seen with LDL treatment during TEM (Supplemental Fig. 4h).

**Figure 5:**
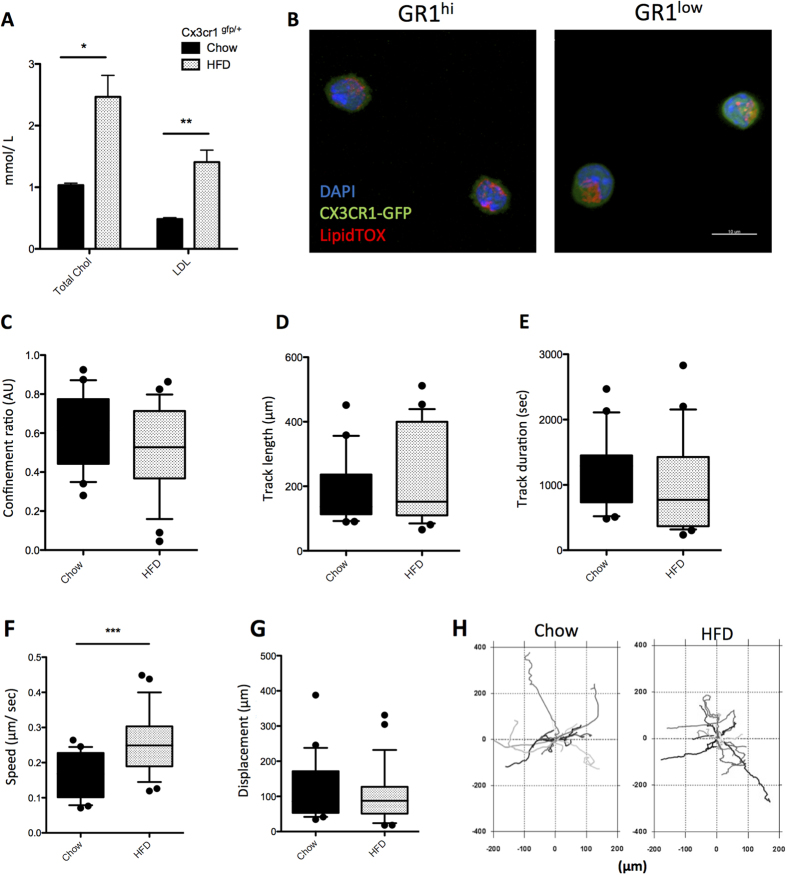
Intravital imaging of monocytes in chow or high fat diet (HFD) fed Cx3cr1^gfp/+^ mice. *Cx3cr1*^gfp/+^ mice were fed chow or HFD for 6 weeks (**A**) Blood total and LDL cholesterol levels of chow vs HFD mice. (n = 4 per group) (**B**) Representative neutral lipid staining of Gr1^hi^ or Gr1^low^ monocytes from *Cx3cr1*^gfp/+^ mice on HFD. Scale bar represents 10 μm. GFP^high^Gr1^low^ monocyte vascular crawling was then assessed by intravital microscopy. (n = 4 per group; n = 20–25 cells per condition) (**C**) Track straightness (**D**) Track length (μm) (**E**) Track duration (seconds) (**F**) Track speed (μm/sec) and (**G**) Track displacement (μm) length of intravascular monocytes during approximately 45 minutes of imaging. (**H**) Track projections of patrolling monocytes from Cx3cr1^gfp/+^ mice fed HFD or chow during ~45 minutes intravital imaging. Error bars show the mean ± SEM. * and ** represents P < 0.05 and P < 0.01 respectively analysed by Mann–Whitney U test.

No defect in monocyte TEM suggests that lipid loading is inhibiting only extravascular migration. To confirm that intravascular monocyte migration is not affected, we performed live intravital imaging of the vasculature of the ear in *Cx3cr1*^gfp/+^ mice fed HFD or chow for 6 weeks. Without further genetic manipulation we were able to significantly increase both total cholesterol and LDL levels after 6 weeks HFD (Fig. 5a), as has been reported by others^38^. This increased neutral lipid loading in blood monocytes (Fig. 5b), similar to *Ldlr*^*−/−*^ mouse on HFD. Corroborating *in vitro* endothelial migration findings, HFD did not cause any clear migratory defect in GR1^low^ monocytes crawling on the vascular endothelium (Fig. 5c–h). While monocytes exhibited a modest increase in speed on HFD (Fig. 5f), this was not accompanied by changes in track displacement, straightness, length or duration (Fig. 5c–e,g,h).

### VLDL modulates cytoskeletal-signaling in monocytes

Given previous work implicating cholesterol in cytoskeletal disruption in macrophages^31^,^32^ and the decreased *RhoA* expression which we observe in the inflamed peritoneum after HFD (Fig. 2f), we hypothesized that neutral lipid loading may be altering cytoskeletal rearrangement in monocytes, thus impairing migration.

**Figure 6:**
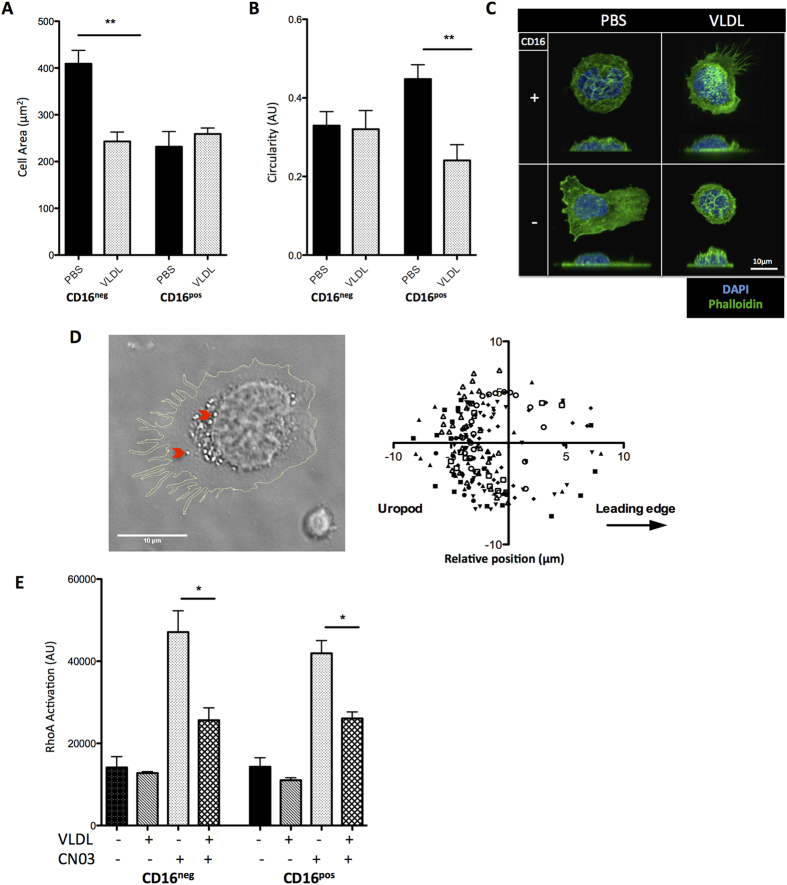
Cytoskeletal dynamics in VLDL treated human monocytes. Human CD16^pos^ and CD16^neg^ monocytes were treated with or without VLDL (100 μg/ml; 2 hrs) and morphology analysed using phalloidin staining. (n = 20 cells per condition). (**A**) Cell area (um^2^) (**B**) circularity and (**C**) representative images of CD16^pos^ and CD16^neg^ monocytes pre-treated with PBS or VLDL. Scale bar = 10 μm. (**D**) Representative spatial localization of lipid-droplets in VLDL treated monocyte, assessed by brightfield microscopy. Red arrows indicate lipid vesicle. Scale bar = 10 μm (**E**) RHOA activation in CD16^pos^ and CD16^neg^ monocytes after treatment with PBS, VLDL or VLDL with or without CN03 (2 μg/ml). (n = 3 donors). Error bars show the mean ± SEM. * and ** represents P < 0.05 and P < 0.01 respectively analysed by Mann–Whitney U test. See also Sup. Fig 4.

Initially, we used fluorescent phalloidin to visualize actin after monocytes had been VLDL treated and allowed to adhere to tissue-culture plastic. When analyzed by confocal microscopy clear effects of VLDL treatment on monocytes were observed, and were distinct between monocyte subsets. While CD16^neg^ cells had a decreased cell spreading after lipid loading resulting in a lower cell area (Fig. 6a,c), CD16^pos^ cells displayed a decreased circularity due to pronounced filopodia-like protrusions (Fig. 6b,c). Interestingly, in some CD16^pos^ polarised monocytes after VLDL treatment, vesicles accumulated within the uropod away from the leading edge (Fig. 6d).

RHOA activation via GTP binding is required for leukocyte chemotaxis^39^ and has previously been implicated in monocyte uropod retraction^33^,^39^ therefore we assessed RHOA activation in response to VLDL and the RHOA activator CN03. VLDL alone had little effect on RHOA-GTP levels, yet when cells were treated with CN03, VLDL significantly impaired RHOA activation in both monocyte subsets (Fig. 6e). We also investigated the activation of the cytoskeletal GTPase CDC42, which is involved in filopodia formation in leukocytes, and the CDC42 and RAC1 effector proteins PAK1 and PAK2, but saw no effect of VLDL or LDL treatment on these two candidates (Supplemental Fig. 4i,j).

## Discussion

We show here that monocytes can be extensively loaded with neutral lipids *in vivo* during high fat diet and *in vitro* following LDL and VLDL treatments. It is unclear whether this is due to endocytosis of the entire lipoprotein, or due to hydrolysis of fatty acids at the cell surface, as hypothesized in smooth muscle cells by Ira Goldberg and colleagues^40^. In peripheral tissues, SR-BI may act synergistically with CD36, heparan sulphate proteoglycans and cell surface lipases to mediate the hydrolysis and uptake of FFAs from triglyceride-rich lipoproteins (TGRL) such as VLDL^41^. While in macrophages the LDL receptor (LDLR) has been implicated in VLDL uptake and trafficking^42^, we believe monocyte lipid loading is independent of LDLR, as lipid uptake occurs in the hypercholesterolemic LDLR knockout mouse. LDLR independent uptake of TGRL would be an interesting study in monocytes.

Monocyte ‘foam cells’ or lipophages have been previously demonstrated in hyperlipidaemic rats^21^, *Ldlr*^*−/−*^ peritoneal macrophages^30^,^43^, *ApoE*^*−/−*^ monocytes during atherosclerosis^22^,^25^, familial hypercholesterolemia patients^18^ and in postprandial monocytes^23^. In some of these studies it is reported that this can lead to monocyte activation exemplified by CD11c expression^22^,^23^. Surprisingly, under our experimental settings we did not observe an obvious monocyte activatory phenotype after neutral lipid accumulation. The disparities are most likely due to the differences in methods of monocyte isolation, treatment dose and time, nuances in lipid species or effects specific to one murine atherosclerosis model, which we did not investigate. Moreover, only a very small fraction of mouse monocytes actually expressed CD11c, which is similar to previous reports (reviewed in^44^), but not by others^22^,^25^. More work is needed to investigate CD11c expression on monocytes in various murine strains under different experimental conditions and the conditions in which lipoproteins may activate monocyte subsets.

Regardless of activation phenotype, a feature of the advanced atherosclerotic plaque is the inhibition of cellular efflux or migration from the plaque^45^,^46^. This led us to explore the effect of lipids on monocyte migration and the role of cytoskeletal genes in monocytes loaded with neutral lipids. We show that *in-vivo* during hyperlipidaemia monocytes become trapped in the omentum and are unable to effectively migratory into the peritoneum during peritonitis. We hypothesise this phenotype is responsible for increased bacterial and fungal pathogenicity reported during hyperlipidaemia^13^,^14^,^15^,^16^. Moreover, we can recapitulate this dysfunctional migration in-vitro using transwell and 2D chemotaxis and chemokinesis assays. Lipid loading significantly inhibited C5a induced migration and polarization. This migratory dysfunction after lipid loading is thought to be extravascular, based on 3 key findings: 1) monocytes are retained in the omentum following peritonitis on HFD 2) luminal endothelial monocyte crawling is not reduced during hypercholesterolemia *in-vivo* and 3) monocyte endothelial adhesion or transendothelial migration is not reduced after lipid treatments *in vitro*. Under our conditions, LDL but not VLDL increased monocyte adhesion to endothelial cells. This is in contrast to a previous report showing increased adhesion after VLDL treatment^47^. The differences are most likely that we used a static adhesion assay, which does not take into account the complex relationship between shear stress and adhesion molecule dynamics^48^ and/or may be dependent on lipoprotein concentration or composition, which we did not investigate.

*RhoA* transcripts were down regulated during HFD peritonitis; therefore we investigated whether lipid loading inhibited monocyte cytoskeletal signaling. Cytoskeletal regulation involves many proteins including the small GTPases RAC, CDC42 and RHO and is a complex and dynamic network that responds to a variety of agonists^49^. A series of phosphorylation events regulate cellular morphology and migration through formation of lamellipodia, filopodia and the extension and retraction of the uropod^49^. In our hands, lamellipodia and filopodia formation is different in each subset after neutral lipid loading with a significant decrease in circulatory index of non-classical monocytes. Interestingly, we also noted an accumulation of vesicles in the uropod in some of the polarised CD16^pos^ non-classical monocytes, which may be involved with inhibition of uropod retraction and impairment of forward cell migration^33^. Given that we did not see subset specific differences in the inhibition of 2D migration towards C5a, we therefore proceeded to investigate cytoskeletal signaling. VLDL treated monocyte subsets exhibit no change in CDC42 activity or phosphorylation of the RAC/CDC42 downstream effector PAK, supporting our hypothesis that this migratory phenotype is not mediated by an increase in membrane cholesterol as described in ABCA1/ABCG1^*−/−*^ macrophages^31^. However we did note a significant decrease in gene expression of RHOA after peritonitis during high fat diet and therefore, we investigated RHOA activity after lipid loading.

Our studies on the RHOA pathway were performed using a direct RHOA activator (CN03), which is based on the catalytic domain of bacterial cytotoxic necrotizing factor toxins^50^. While there was a significant inhibition of RHOA activity in monocytes after VLDL treatment, this was not subset specific. The inhibition of RHOA activity by neutral lipids may indicate a direct binding to RHO GTPase by a neutral lipid moiety. Overall we believe that there are multiple cytoskeletal signals involved in monocyte lipid accumulation, some of which are subset specific and mediate changes in morphology, while others potentially affect RHOA downstream signaling in all monocytes. More work is needed to dissect these multiple cues and the mechanisms that dictate subset specific phenotypes.

During atherosclerosis others have already shown that dyslipidaemia is able to perturb macrophage migration through modulation of cytoskeletal rearrangement by free cholesterol in the cell membrane^31^,^32^,^51^, perhaps contributing to increased macrophage plaque retention^19^. For example, a paper by the Tall group showed that the defect in migration was mediated by RAC1 and involved the cholesterol transporter ABCA/G1^31^. However our observations are independent from these on a number levels: 1) Neutral lipid and not unesterified cholesterol accumulate in vesicles following lipoprotein exposure 2) we observe no change in membrane cholesterol levels in LDL/VLDL treated CD16^pos^ monocytes, which have a migratory phenotype. This confirms Pagler *et al*.^31^, describing no effect of LDL treatment on membrane cholesterol accumulation in wild-type macrophages. 3) We show migratory dysfunction is only dependent on RHOA and not PAK1 (downstream of RAC1) or CDC42. These different observations between monocytes and macrophages highlight the discrete functional independence of mononuclear phagocytes during dyslipidaemia. Furthermore, we hypothesise that independent mechanisms govern the effects of unesterfied cholesterol efflux versus neutral lipid droplet accumulation in these cells.

In summary we demonstrate the effects of hyperlipidaemia on the migration and cytoskeletal regulation of monocyte subpopulations. This work extends the function of ‘foamy monocytes’ or lipophages beyond their recently demonstrated role in delivering lipid during early atherogenesis^25^. Although our work may highlight a mechanism for monocyte retention in the atherosclerotic plaque, perhaps more importantly there may be broader implications for dyslipidaemia- induced monocyte immunosuppression. Our study emphasises the role of dyslipidaemia in infection and innate immunity and the need to further study the effects of lipid accumulation in both blood and tissue compartments.

## Additional Information

**How to cite this article**: Jackson, W. D. *et al*. Very-low and low-density lipoproteins induce neutral lipid accumulation and impair migration in monocyte subsets. *Sci. Rep*. **6**, 20038; doi: 10.1038/srep20038 (2016).

## Supplementary Material

Supplementary Information
