# Supplementary material for: Very-low and low-density lipoproteins induce neutral lipid accumulation and impair migration in monocyte subsets
Source: Sci Rep. 2016 Jan 29;6:20038. doi: 10.1038/srep20038 (PMC4731823; doi:10.1038/srep20038)
Supplement: Supplementary Information [file srep20038-s1.pdf]

Supplemental Figures and Methods

Very-low and low-density lipoproteins induce neutral lipid accumulation and impair migration in monocyte subsets

William D Jackson, Tobias W Weinrich and Kevin J Woollard

SUPPLEMENTAL FIGURE LEGENDS

Supplemental Figure 1: FACS phenotyping of blood leukocytes from *Ldlr*$^{-/-}$ mice. Blood monocytes from *Ldlr*$^{-/-}$ mice fed chow or HFD for 6 weeks were analyzed by flow cytometry. n=4 mice per group (**A**) GR1^{hi} and GR1^{low} monocyte CD11b median fluorescence intensity (MFI), (**B**) I-A MFI and (**C**) GR1 MFI. **(D)** Percentage of GR1^{hi} and GR1^{low} monocytes that are CD36^{pos} on chow or HFD. Blood neutrophils were analyzed for (**E**) SSC and (**F**) CD11b MFI. Error bars show the mean±SEM. ** represents P<0.01, analysed by Mann–Whitney U test.

Supplemental Figure 2: Mouse blood monocyte and peritoneal monocyte/ macrophage gating strategy and tracking during peritonitis. (**A**) Blood monocyte subpopulations were gated as follows: debris and platelets were excluded using FSC and SSC, representing size and granularity respectively. Single cells were selected using ratio of SSC area to width, and of those CD115^{+} CD11b^{+} cells were gated as Gr1^{hi} or Gr1^{low} monocytes. (**B**) Peritoneal monocytes/ macrophages were gated as follows: debris and platelets were excluded using FSC and SSC, representing size and granularity respectively. Single cells were selected using ratio of SSC area to width, and of those CD115^{+} CD11b^{+} cells were gated as infiltrating monocytes/ macrophages. **(C)** CD115^{neg} CD11b+ GR1^{high} granulocytes /ml peritoneal lavage, 72 hours post-Thio, from the experiment displayed in **Figure 2**. IV injection of 1µm latex beads was used to track monocyte migration out of the blood during peritonitis in *Ldlr*$^{-/-}$ mice with or without HFD. (n=4 mice per group) (**D**) % of blood Gr1^{hi} or Gr1^{low} monocytes which were bead^{pos} post- bead injection. (**E**) Median bead fluorescence intensity of bead^{+} peritoneal monocytes/ macrophages. (**F**) Representative plots showing latex bead positive populations gated from peritoneal CD115^{+} CD11b^{+} monocytes/ macrophages after 72 hours thioglycollate peritonitis. This is quantified as (**G**), bead positive monocytes per ml of peritoneal lavage on chow or HFD. Error bars show the mean±SEM.

Supplemental Figure 3: Human monocyte surface marker expression, apoptosis and phagocytosis after LDL and VLDL treatment. (A-H) For all experiments, CD16^{pos} and CD16^{neg} human monocytes were first incubated with and without 100µg/ml LDL or VLDL for 2 hours. After LDL treatment: cell surface (**A**) HLA-DR MFI (**B**) CD14 MFI (**C**) CD16 MFI and (**D**) CD11c MFI. Corresponding data for VLDL shown in (**E-H**). (**I**) % of cells which are apoptotic as assessed by annexin-V staining. (**J-L**) Cells were incubated with carboxylated 1µm latex beads to assess phagocytosis (**J**) Representative histogram of latex bead gating. (**K**) % of cells which are bead^{neg} or (**L**) which have phagocytosed >4 beads after 1 hour incubation. n=3 donors in triplicate. Error bars show the mean±SEM.

Supplemental Figure 4: Further effects of LDL and VLDL treatment on monocyte migration. (**A**) Transwell migration of CD16^{pos} and CD16^{neg} monocytes to C5a (250ng/ml) with or without VLDL pre-treatment (2hrs; 100ug/ml). n=3 donors. (**B**) Track projections from monocytes with or without VLDL pre-treatment in a 2D real-time chemokinesis assay with C5a stimulation. Tracks were analyzed for (**C**) displacement (µm) (**D**) speed (µm/sec) (**E**) confinement ratio (AU). n=50-160 cells per condition. (**F**) Adhesion of CD16^{pos} and CD16^{neg} monocytes to HUVECS from only monocytes pre-treated with LDL (2hrs; 100ug/ml), normalised to CD16^{neg} PBS treated (n= 4 donors), (**G**) Same as (F) with only HUVECS treated with VLDL. (**H**) Transmigration of CD16^{pos} and CD16^{neg} monocytes pre-treated with (2hrs; 100ug/ml) or without LDL through TNF activated HUVEC: cells per field, normalised to CD16^{neg} PBS treated. (n=4 donors). (**I-J**) Human CD16^{pos} and CD16^{neg} monocytes treated with LDL or VLDL (100ug/ml) for 2 hours were assessed for (**I**) CDC42 activity using ELISA or (**J**) PAK1/2 phosphorylation using Western blot, n=3 donors. A representative blot is shown for PAK1/2 phosphorylation. Error bars show the mean±SEM. * and *** represents P<0.05 and P<0.001 respectively analysed by Mann–Whitney U test.

Supplemental Figure I

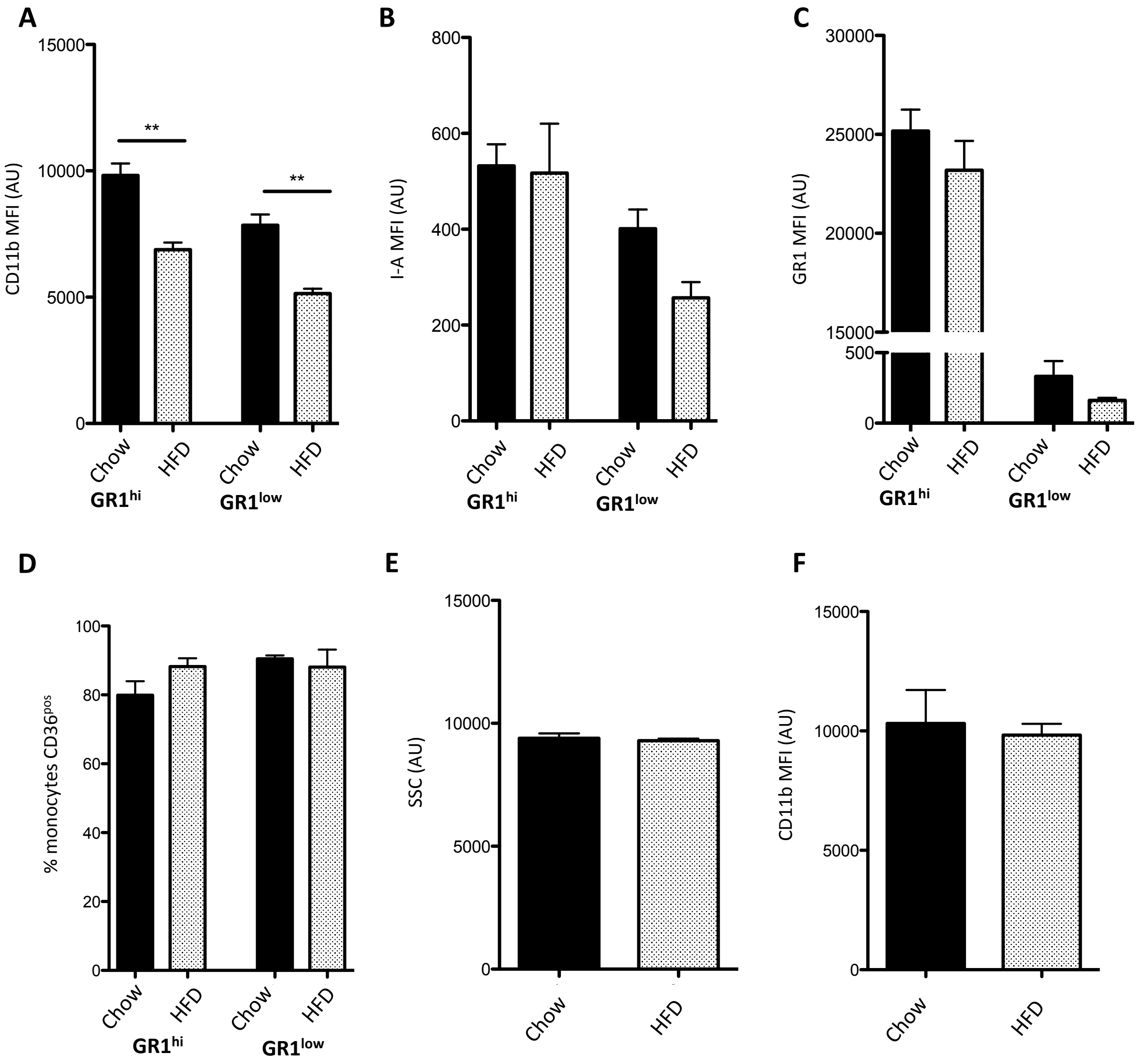

Supplemental Figure 2

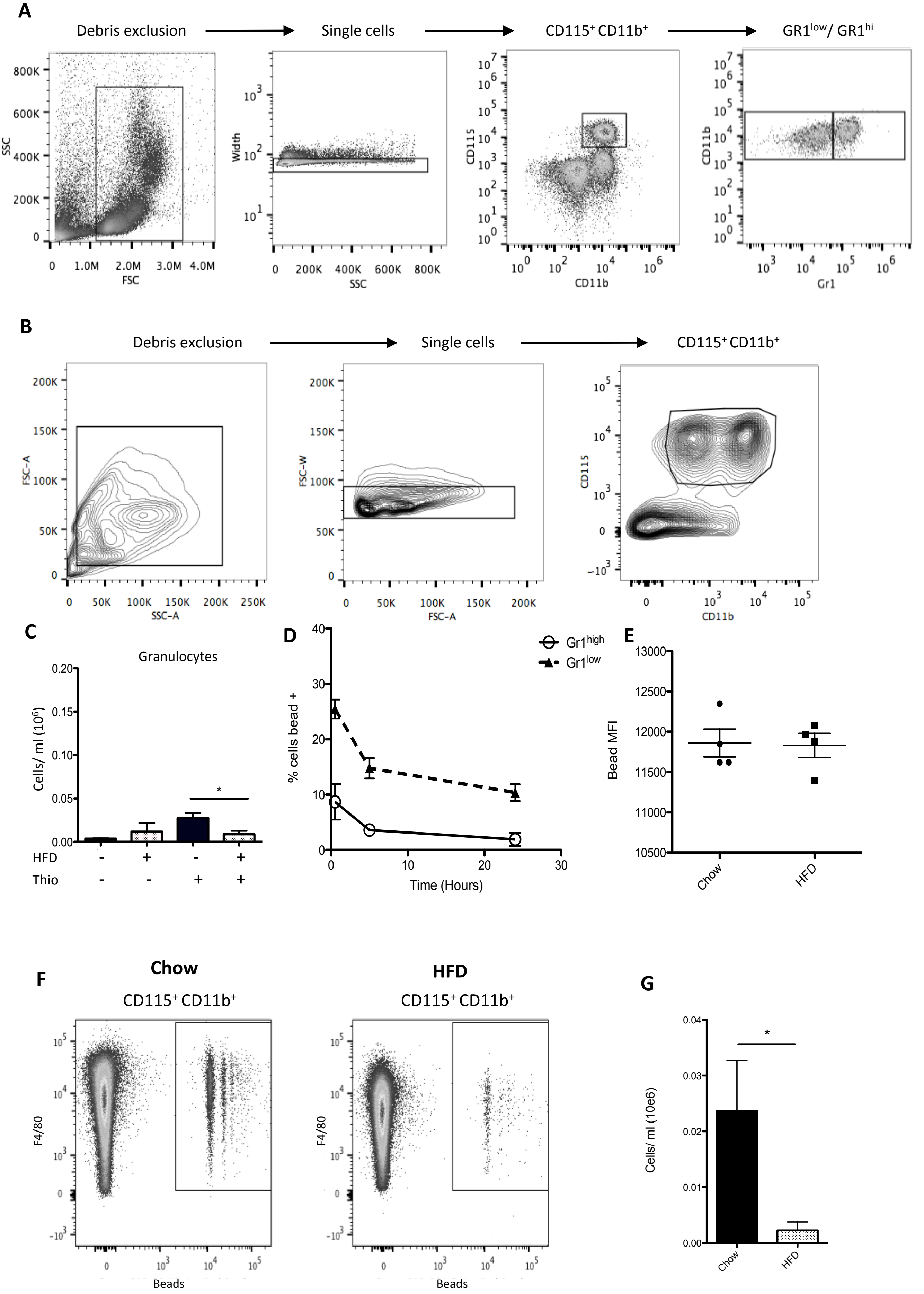

Supplemental Figure 3

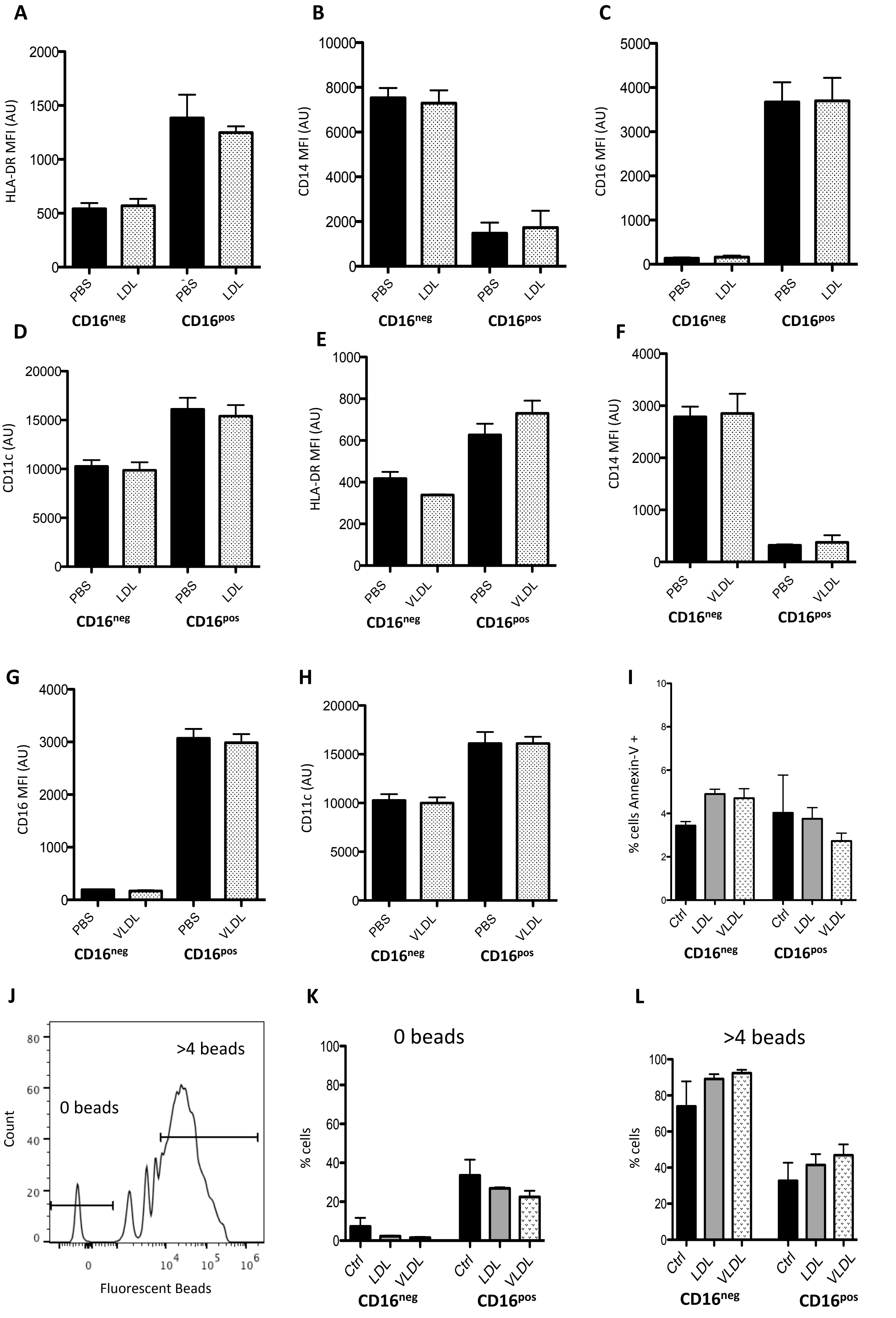

Supplemental Figure 4

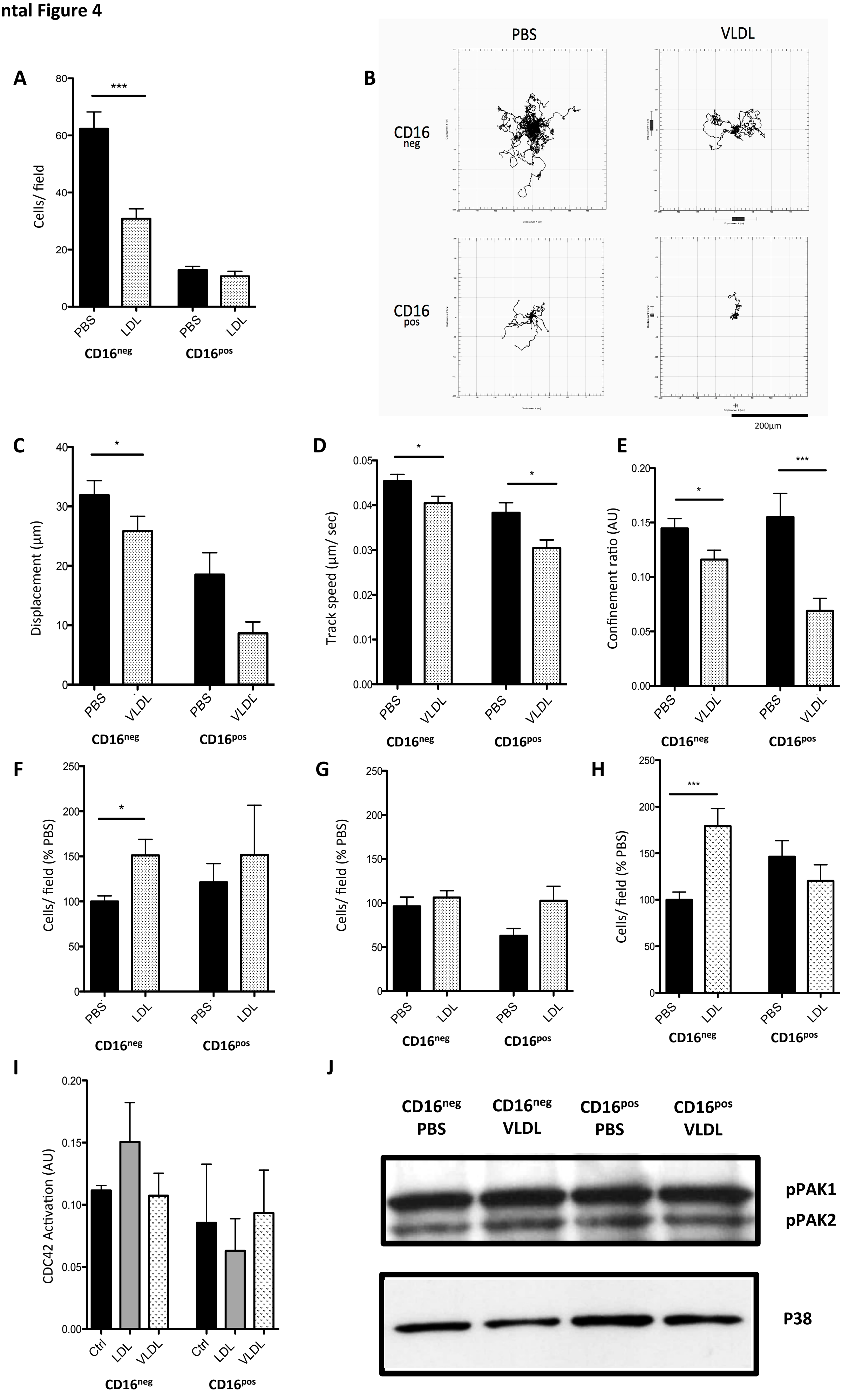

Supplemental MATERIALS AND METHODS

Mice

C57BL/6 mice were purchased from Charles River. C57BL/6.LDLR-deficient mice (*Ldlr*$^{-/-}$) and B6.129P-*Cx3cr1*^{tm1Litt}/J (*Cx3cr1*$^{gfp/gfp}$) were purchased from Jackson Laboratory (Bar Harbor, USA). All animals were housed in individually ventilated cages and handled in accordance with institutional guidelines and procedures approved by the UK Home Office. Mice were maintained on a high fat (HFD) or standard chow diet for 6-16 weeks, as specified in individual experiments below. High fat diet contained 15% cocoa butter and 1% corn oil, resulting in a total fat and cholesterol content of 16% and 0.25% respectively (Arie Blok Animal Nutrition).

Peritonitis model

Ldlr$^{-/-}$ mice were maintained on HFD or chow for 16 weeks. To induce peritonitis, mice were injected intraperitoneally (IP) with 1ml sterile 4% thioglycollate medium. After 72 hours, mice were culled by exposure to rising concentration of CO^2 and the peritoneal cavity was lavaged with 10ml ice-cold PBS. Cells were counted on a hemocytometer and washed in PBS. Approximately 3 x 10^5 cells were stored in Tri-Reagent (Sigma Aldrich) for RNA extraction, the remainder were stained in PBS-0.5% BSA for flow cytometric analysis in a saturating concentration of anti-CD16/32 (2.4G2) using combinations of the following antibodies: anti-CD11c (N418), anti-CD36 (REA262), anti-CD115 (AF598), anti-CD45 (30-F11) (eBioscience), anti-CD11b (M1/70), anti-GR1 (RB6-8C5) anti-F4/80 (BM8) (BD Biosciences). Monocytes were defined as CD115^{pos} CD11b^{pos} SSC^{int}.

In some experiments, omental tissue was harvested by blunt dissection into HBSS and the weight recorded. Tissue was washed and digested using Liver Digestion Media (Gibco) for 15 minutes at 37°C. After washing to obtain a single-cell suspension, cells were counted and stained for flow cytometry as detailed above.

To track monocyte migration from the blood into the peritoneum, mice were injected IV with 100µl 1µm red latex beads, (Invitrogen). Beads were injected 4 hours prior to IP thioglycollate injection and the peritoneal lavage was collected at 72 hours for flow cytometry as detailed above.

Monocyte purification

Peripheral blood from healthy volunteers was collected by venepuncture into EDTA vacutainers after informed consent. Human peripheral blood mononuclear cells (PBMCs) were isolated by density centrifugation using Lymphoprep (Stem Cell) according to manufacturer instructions. Total monocytes were obtained by magnetic depletion of lineage positive leukocytes with the Monocyte Isolation Kit II, with the addition of an anti-

CD61-Biotin antibody to the depletion antibody cocktail to eliminate platelet contamination (Miltenyi). Monocytes were separated into CD16^{pos} and CD16^{neg} populations by magnetic positive selection using anti-CD16 MicroBeads (Miltenyi). Population purity was confirmed by flow cytometry. In some experiments, whole leukocytes were isolated by red blood cell lysis using a hypotonic lysis buffer (dH_2O, NH_4Cl, $NaHCCO_3$, EDTA). Surface protein expression was assessed by flow cytometry using anti- HLA-DR (TU36), anti- CD16 (3G8) and anti- CD14 (M5E2) (BD Biosciences) and anti-CD11c (Bu15).

Mouse monocytes were purified from peripheral blood samples obtained by tail vein venipuncture or cardiac puncture under terminal anesthesia. Peripheral blood mononuclear cells (PBMCs) were isolated by density centrifugation using Lymphoprep (Stem Cell) as above and monocyte subpopulations were obtained by FACS according to the gating strategy in Supplemental Figure 2A, using the antibodies listed above. Surface protein expression was assessed by flow cytometry.

Quantifying LDL/VLDL modification

Human LDL or VLDL were purchased from Biomedical Technologies, Inc. Vials were opened upon receipt and left for approximately 1 week at 4°C before use. Lipid modification was measured via the absorbance of conjugated dienes at 234nm as previously described [1] using a NanoDrop 2000 spectrophotometer (Thermo Scientific).

Monocyte adhesion to endothelium

Human umbilical venous endothelial cells (HUVECs) were a kind gift from Professor Justin Mason and were isolated and cultured as previously described [2]. Briefly, HUVECs were seeded into collagen-I (Sigma-Aldrich) coated 0.4µm chamber slides (IBIDI) at 2 x 10^6/ml and allowed to adhere 30 minutes at 37°C. Cells were washed with complete M199 and left to reach confluence for 24 hours at 37°C. In separate experiments, HUVECs or human monocytes were treated with 100µg/ml LDL or VLDL for 2 hours at 37°C. Cells were washed and monocytes were stained with NucBlue (Life Technologies) and added to the endothelium at 1 x 10^6/ml for 30 minutes. Co-cultures were fixed in 4% PFA and washed to remove non-adherent monocytes. Slides were imaged using a 20X objective on a Leica SP5 confocal microscope and nuclei were counted using ImageJ analysis software.

Transwell chemotaxis assay

Purified human CD16^{pos} and CD16^{neg} blood monocytes were incubated in DMEM for 2 hours at 37°C with or without 100µg/ml LDL or VLDL (Biomedical Technologies, Inc). 1 x 10^5 cells per well were seeded into 3µm-pore transwell

inserts (Corning), using a chemoattractant gradient of 250ng/ml recombinant human C5a (RND Systems) and incubated at 37°C for 2 hours. Non-adherent cells were removed and the transwell inserts were fixed in 4% paraformaldehyde (PFA). Non-migrated cells were removed using a cotton bud before the membranes were excised and mounted on microscope slides in Vectashield mounting media containing DAPI (Vector Labs). Slides were imaged using a 20X objective on an Olympus BX51 widefield fluorescence microscope and nuclei were counted using ImageJ analysis software.

For endothelial transmigration experiments, transwell inserts were coated with Type I collagen (Sigma Aldrich) overnight, seeded with HUVECs at 1 x 10^5 cells/cm^2 and allowed to reach confluence for 24 hours. HUVECs were activated with 2ng/ml TNF-α (PeproTech) for 3 hours. Monocytes were labeled with CFSE for 30 minutes to allow for distinction from endothelial cells, washed and seeded into transwells as described above. Transmigrated monocytes were imaged as described above.

2D real-time migration assay

Purified human CD16^{pos} and CD16^{neg} blood monocytes were re-suspended at 3 x 10^6 cells/ml in DMEM-0.5% BSA and seeded into IBIDI 2D chemotaxis chambers (IBIDI) for 1 hour at 37°C with or without 100µg/ml LDL or VLDL (Biomedical Technologies, Inc). CD16^{pos} monocytes were labelled for imaging using an AlexaFluor-647 conjugated anti-CD16 antibody (3G8, Biolegend) 1/25 concentration. For chemotaxis experiments, a gradient of 250ng/ml human recombinant C5a was established on one side of the chamber according to manufacturer instructions. For chemokinesis experiments, C5a was added directly to the cell channel to achieve uniform distribution. Slides were allowed to equilibrate for 30 minutes before imaging on a Leica SP5 confocal microscope using a 10x/0.4 objective and a 37°C environmental chamber.

qPCR

RNA was extracted by acid guanidinium thiocyanate-phenol-chloroform extraction using Tri-Reagent (Sigma Aldrich). cDNA was synthesized by reverse transcription with SuperScript III Reverse Transcriptase (Life Technologies). The qPCR reaction was performed on an Eppendorf Mastercycler Ep system using Sensimix SYBR (Bioline). Input cDNA was quantified using a standard curve generated with serial dilutions of pooled samples and normalized to the mean values of housekeeping GAPDH and 18s-rRNA. All primers were ordered as custom oligonucleotides from Sigma Aldrich and are listed below.

Target	Forward	Reverse

IL1B	CAACCAACAAGTGATATTCTCC	GATCCACACTCTCCAGCTGCA
TNF	CATCTTCTCAAAATTCGAGTGACAA	TGGGAGTAGACAAGGTACAAC CC
IL10	CAGCCGGGAAGACAATAACTG	CCGCAGCTCTAGGAGCATG
GSN	CTCTGGACCACCACCTCATT	GTTCAGGGCTTTGAGTCGTC
CDC42	CCCATCGGAATATGTACCAACTG	CCAAGAGTGTATGGCTCTCCA C
RAC1	GAAAGAGATCGGTGCTGTCAA	CAACAGCAGGCATTTTCTCTT
RHOA	AGCTTGTGGTAAGACATGCTTG	GTGTCCCATAAAGCCAACTCTA C
GAPDH	AGGTCGGTGTGAACGGATTTG	TGTAGACCATGTAGTTGAGGTC A
18S	CTGGAGCCTGTTTTGCTTCTG	TGAGATGGACTGTCGTCGGAT G
CX3CR1	GAGTATGACGATTCTGCTGAGG	CAGACCGAACGTGAAGACGAG
CXCL10	CTCATCCTGCTGGGTCTGAG	CCTATGGCCCTCATTCTCAC

Fluorescence microscopy:

Monocytes (1.5x10^{6}/ml) were seeded into tissue-culture treated 0.4μm chamber slides (IBIDI) in DMEM and allowed to adhere 30 minutes at 37°C. Adherent cells were immediately fixed in 4% PFA for 15 minutes and washed with 0.1M glycine. For neutral lipid staining, LipidTox-Green (Life Technologies) was diluted 1/200 in PBS. To image the actin cytoskeleton cells were stained with phalloidin- AlexaFluor 488 (Life Technologies), 1/40 in PBS. Cells were mounted in DAPI-Vectashield (Vector Labs) and imaged using either a Zeiss AxioObserver widefield or a Leica SP5 confocal microscope with a 63x/1.4 objective. Images were analysed using Imaris software (Bitplane) or ImageJ, as indicated.

Intravital imaging

Cx3cr1$^{gfp/+}$ mice were fed chow or HFD for 6 weeks and were 10-12 weeks of age when used for imaging. Blood lipid profiles were generated by automated analysis using an Abbott Architect ci8200 (Abbott Diagnostics). Intravital imaging of the ear dermis was performed as previously described [3]. Briefly, mice were anesthetized using a cocktail of fentanyl/fluanisone and midazolam injected intraperitoneally and were maintained at 37°C with oxygen supplementation. The ear to be imaged was taped to the center of the coverslip and 80μL of TRITC conjugated 70kDa dextran (70μM) was injected intravenously. Light was generated from 488-nm and 562-nm lasers, and emitted light signal was detected to generate two colour 8-bit images, using a 10x/0.4 objective on a Leica SP5 confocal microscope. Images were analysed

using Imaris software (Bitplane). Briefly, dextran signal was used to select only intravascular cells for tracking and cells were automatically selected based on the quality and intensity of their GFP signal. Cells were tracked using the inbuilt autoregressive model based algorithm and tracks were manually verified for accuracy. In order to analyse intravascular patrolling, cells that were present for less than 240 seconds were excluded.

Phagocytosis

Purified human peripheral blood mononuclear cells were incubated at 5 x 10^6/ml in DMEM for 2 hours at 37°C with or without 100µg/ml LDL or VLDL (Biomedical Technologies, Inc). To assess phagocytosis, cells were treated 1/100 with 1µm carboxylated fluorescent yellow-green latex beads (Sigma Aldrich) for 1 hour at 37°C. Cells were washed twice and stained for flow cytometry using anti- HLA-DR (TU36), anti- CD16 (3G8) and anti- CD14 (M5E2).

Apoptosis

Purified human peripheral blood mononuclear cells were incubated at 5 x 10^6/ml in DMEM for 2 hours at 37°C with or without 100µg/ml LDL or VLDL (Biomedical Technologies, Inc). Cells were washed and stained for apoptosis using an anti-Annexin V antibody at a concentration of 1/20 according to manufacturer instructions (eBioscience). Cells were washed again and stained for flow cytometry using anti- HLA-DR (TU36), anti- CD16 (3G8) and anti- CD14 (M5E2).

RHOA and CDC42 activation assays

Human $CD16^{pos}$ and $CD16^{neg}$ primary monocytes (0.5 x 10^6) were treated with combinations of 100 µg/mL LDL/ VLDL and 2µg/ml RHOA activator CN03 (Cytoskeleton, Inc.) for 2 hours in DMEM at 37°C. Cells were lysed with supplied lysis buffer and lysates clarified by centrifugation at 4˚C (14000 g, 3 min). Protein concentration for each sample was determined using the BCA assay (Thermo Fisher Scientific) and remaining lysates were snap-frozen in liquid nitrogen for storage at -80˚C. RHOA activity in human primary monocytes was measured using a luminescence-based G-LISA RHOA activation kit according to manufacturer instructions (Cytoskeleton, Inc.). Chemiluminescence was measured at 445 nm 5 min after the HRP substrate was added, using a FluoStar Galaxy instrument (BMG Labtechnologies, NC, U.S.A.). CDC42 activity was assessed in cells treated and processed as above using a colorimetric-based G-LISA kit according to manufacturer instructions (Cytoskeleton, Inc.) and absorbance at 562 nm was measured using a Biotek ELX 800 microplate reader (Bio-Tek Instruments).

Western blot

Monocytes were lysed on ice in 70µL of lysis buffer (50 mM NaCl, 250 mM Tris-HCL, 1% NP-40) supplemented with phosphatase inhibitors (5 mM EDTA, 50 mM NaF, 1 mM Na_3VO_4) and protease inhibitor cocktail (Sigma-Aldrich) for 30 minutes. Proteins were separated by 15% SDS-PAGE (sodium dodecyl sulfate polyacrylamide gel electrophoresis) and transferred onto nylon membranes (GE Healthcare). Membranes were blocked in TBS containing 5% BSA and 0.1% Tween 20 and incubated overnight with anti-phospho-PAK1/2/3 (Novus Biologicals) or anti-p38 (SC535, Santa Cruz Biotechnology). Protein was detected with horseradish peroxidase-conjugated secondary antibodies (Cell Signaling Technology) in combination with Amersham ECL Western Blotting Reagants (GE Healthcare).

Statistics

Experimental data is presented as mean +/- standard error of the mean (SEM). Populations were compared using a two-tailed Mann–Whitney U test to avoid assumptions of parametric distribution. $P<0.05$ was considered significantly different.

Supplemental References:

1. Esterbauer H, Striegl G, Puhl H, Rotheneder M. Continuous monitoring of in vitro oxidation of human low density lipoprotein. *Free Radic. Res. Commun.* 1989;6(1):67–75.

2. Mason JC, Yarwood H, Sugars K, Morgan BP, Davies KA, Haskard DO. Induction of decay-accelerating factor by cytokines or the membrane-attack complex protects vascular endothelial cells against complement deposition. *Blood* 1999;94(5):1673–1682.

3. Carlin LM, Auffray C, Geissmann F. Measuring Intravascular Migration of Mouse Ly6Clow Monocytes In Vivo Using Intravital Microscopy. *Current Protocols in Immunology* 2013:14.33.1–14.33.16. doi:10.1002/0471142735.im1433s101.
